# High-Affinity Chemotaxis to Histamine Mediated by the TlpQ Chemoreceptor of the Human Pathogen Pseudomonas aeruginosa

**DOI:** 10.1128/mBio.01894-18

**Published:** 2018-11-13

**Authors:** Andrés Corral-Lugo, Miguel A. Matilla, David Martín-Mora, Hortencia Silva Jiménez, Noel Mesa Torres, Junichi Kato, Akiko Hida, Shota Oku, Mayte Conejero-Muriel, Jose A. Gavira, Tino Krell

**Affiliations:** a Department of Environmental Protection, Estación Experimental del Zaidín, Consejo Superior de Investigaciones Científicas, Granada, Spain; b Department of Molecular Biotechnology, Graduate School of Advanced Sciences of Matter, Hiroshima University, Higashi-Hiroshima, Hiroshima, Japan; c Laboratory of Crystallographic Studies, IACT, (CSIC-UGR), Armilla, Spain; University of Missouri-Columbia; Institut Pasteur

**Keywords:** *Pseudomonas aeruginosa*, chemotaxis, histamine

## Abstract

Histamine is a key biological signaling molecule. It acts as a neurotransmitter in the central and peripheral nervous systems and coordinates local inflammatory responses by modulating the activity of different immune cells. During inflammatory processes, including bacterial infections, neutrophils stimulate the production and release of histamine. Here, we report that the opportunistic human pathogen Pseudomonas aeruginosa exhibits chemotaxis toward histamine. This chemotactic response is mediated by the concerted action of the TlpQ, PctA, and PctC chemoreceptors, which display differing sensitivities to histamine. Low concentrations of histamine were sufficient to activate TlpQ, which binds histamine with an affinity of 639 nM. To explore this binding, we resolved the high-resolution structure of the TlpQ ligand binding domain in complex with histamine. It has an unusually large dCACHE domain and binds histamine through a highly negatively charged pocket at its membrane distal module. Chemotaxis to histamine may play a role in the virulence of P. aeruginosa by recruiting cells at the infection site and consequently modulating the expression of quorum-sensing-dependent virulence genes. TlpQ is the first bacterial histamine receptor to be described and greatly differs from human histamine receptors, indicating that eukaryotes and bacteria have pursued different strategies for histamine recognition.

## Abstract (precis)

Genome analyses indicate that many bacteria possess an elevated number of chemoreceptors, suggesting that these species are able to perform chemotaxis to a wide variety of compounds. The scientific community is now only beginning to explore this diversity and to elucidate the corresponding physiological relevance. The discovery of histamine chemotaxis in the human pathogen Pseudomonas aeruginosa provides insight into tactic movements that occur within the host. Since histamine is released in response to bacterial pathogens, histamine chemotaxis may permit bacterial migration and accumulation at infection sites, potentially modulating, in turn, quorum-sensing-mediated processes and the expression of virulence genes. As a consequence, the modulation of histamine chemotaxis by signal analogues may result in alterations of the bacterial virulence. As the first report of bacterial histamine chemotaxis, this study lays the foundation for the exploration of the physiological relevance of histamine chemotaxis and its role in pathogenicity.

## INTRODUCTION

Bacteria possess different types of signal transduction systems that enable them to adapt to changes in environmental cues. In addition to one- and two-component signal transduction systems, chemosensory pathways play an important role in this process (1–3). In a canonical chemosensory pathway, signaling is initiated by the binding of signal molecules to the chemoreceptor ligand binding domain (LBD), which in turn modulates the autophosphorylation activity of the CheA histidine kinase and the transphosphorylation of the CheY response regulator, which ultimately triggers pathway output (2). While most chemoreceptors mediate chemotaxis, some also carry out alternative cellular functions, such as modulating c-di-GMP levels or type IV pilus-based motility (4–6).

Escherichia coli is the traditional model organism for the study of chemoreceptor-based signaling processes (7). It has 5 chemoreceptors, of which 4 contain a periplasmic 4-helix bundle LBD. Importantly, these chemoreceptors bind signals either directly or in complex with a periplasmic ligand binding protein. E. coli has a single chemosensory cascade that mediates chemotaxis primarily toward sugars, amino acids, or dipeptides (7, 8).

More recently, chemoreceptor-based signaling has been studied in an array of bacteria with different lifestyles (9). The existing data suggest that the typical number of chemoreceptor genes in bacteria, which can reach as high as 80, is much higher than in E. coli (10). Furthermore, sequence analyses indicate that chemoreceptors comprise more than eighty different LBD types (11). The most abundant of these are CACHE-type LBDs, which are present in either the monomodular (sCACHE) or bimodular (dCACHE) form (12). The large number of chemoreceptor genes and the diversity of LBD types suggest that bacteria can respond to a wide variety of signal molecules. The scientific community is now beginning to explore this diversity and to elucidate the corresponding physiological relevance.

Pseudomonads are important model organisms for the study of chemoreceptor function (13, 14), and the strains Pseudomonas putida KT2440 and Pseudomonas aeruginosa PAO1 have been well studied and characterized (11). The former strain is a nonpathogenic soil bacterium with a saprophytic lifestyle (15). In contrast, P. aeruginosa strains are among the most virulent opportunistic human pathogens and the leading cause of nosocomial infections, particularly in immunocompromised, cancer, burn, and cystic fibrosis patients (16).

Strains KT2440 and PAO1 have similar numbers of chemoreceptor genes: 27 and 26, respectively. The function and the corresponding ligand profiles have been established for approximately ten receptors in each strain (11, 17). Among the functionally annotated KT2440 chemotaxis receptors are several for different organic acids (18), purines (19), proteinogenic amino acids (20), and gamma-aminobutyric acid (GABA) (21). In addition, the McpU chemoreceptor of this strain was the first chemoreceptor identified that responded to the polyamines putrescine, spermidine, and cadaverine (20, 22). In contrast, PAO1 chemotaxis to proteinogenic amino acids and GABA is mediated by three paralogous receptors, namely, PctA, PctB, and PctC (23, 24). Additionally, this strain has two receptors for inorganic phosphate (25, 26) as well as receptors for malate (27, 28), α-ketoglutarate (29), and chloroethylenes (30). P. aeruginosa is also attracted to the plant hormone ethylene, and it was shown that the deletion of the gene encoding the TlpQ chemoreceptor abolished ethylene chemotaxis (31).

In this study, we provide the first report of bacterial chemotaxis toward histamine. This compound is produced by different animal tissues and is secreted by some bacteria (32). Histamine is a signal molecule with multiple functions. It is an aminergic neurotransmitter of the central and peripheral nervous systems, and it is involved in numerous biological processes (33). It is also a key modulator of local immune responses by mediating the effects on many cell types such as antigen-presenting cells, natural killer cells, and epithelial cells, as well as T and B lymphocytes (34). Bacteria have been shown to impact histamine function. For example, bacterial respiratory tract infections stimulate neutrophils to release histamine (35, 36). Also, it was shown that infection by PAO1 greatly increased neutrophil histamine content and secretion but did not alter histamine production in mast cells, which are the classical histamine reservoirs (36). Furthermore, it has been shown that histamine might play divergent roles in the immune response: it has been implicated in mediating the defense against infection (37) as well as increasing the susceptibility to infection (38). While there has been preliminary evidence that histamine is a signal molecule for bacteria, the underlying mechanisms remain largely unknown (39). The present study provides important insight into the molecular mechanisms that permit bacteria to sense and respond to histamine.

## RESULTS

### Identification of histamine and additional polyamines as novel ligands for the P. putida KT2440 McpU chemoreceptor.

**FIG 1:**
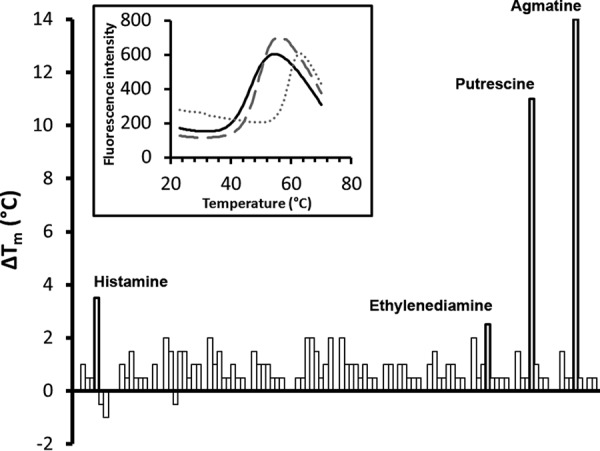
Thermal shift assays of P. putida KT2440 McpU-LBD against a library of ligands. Shown are the individual *T_m_* changes caused by 95 compounds (Biolog array PM3B) that can serve as nitrogen sources. The inset shows the unfolding curves of McpU-LBD when free from ligand (continuous line) and in the presence of agmatine (dotted line) and histamine (dashed line).

By screening 190 compounds for binding to the purified McpU-LBD, we previously found that McpU binds to and mediates chemotaxis to putrescine, cadaverine, and spermidine (20). In the present study, we extended this screening to include 285 additional compounds. These compounds were mostly bacterial nitrogen, phosphorous, and sulfur sources (see Materials and Methods). We used a thermal shift assay to monitor changes in the midpoint of protein unfolding (*T_m_*) caused by ligand binding (40). In the absence of ligand, McpU-LBD had a *T_m_* of 46.5°C. Of the 95 nitrogen sources screened (Biolog plate PM3B), three additional compounds—agmatine, ethylenediamine, and histamine—caused *T_m_* increases greater than 2°C (Fig. 1).

Using isothermal titration calorimetry (ITC), we found that all three compounds bind to McpU-LBD (see Fig. S1A in the supplemental material). Very tight binding was observed for agmatine with a *K_D_* (equilibrium dissociation constant) in the nanomolar range, whereas histamine and ethylenediamine bound with much lower affinities (Table 1). It should be noted that of these three new McpU ligands and the previously identified ligands (i.e., putrescine, cadaverine, and spermidine), all except for histamine are polyamines (Fig. S1B).

**TABLE 1:** Thermodynamic parameters for the binding of ligands to McpU-LBD and TlpQ-LBD as derived from ITC experiments^*a*^

Compound	McpU-LBD		TlpQ-LBD		*K_D_* McpU-LBD/*K_D_* TlpQ-LBD
	*K_D_* (µM)	Δ*H* (kcal · mol^−1^)	*K_D_* (nM)	Δ*H* (kcal · mol^−1^)	
Putrescine	2 ± 0.1^*b*^	−15 ± 0.5	134 ± 12	−6.8 ± 0.3	15
Cadaverine	22 ± 2^*b*^	−15.5 ± 0.5	150 ± 4	−6.0 ± 0.1	147
Spermidine	4.5 ± 0.4^*b*^	−4.3 ± 0.3	56 ± 4	−4.6± 0.4	80
Agmatine	0.48 ± 0.02	−14.5 ± 0.2	150 ± 9	−5.4 ± 0.1	3
Ethylenediamine	39 ± 4	−9.7 ± 0.5	1,710 ± 180	−6.3 ± 0.6	23
Histamine	26 ± 2	−2.6 ± 0.3	639 ± 27	−9.1 ± 0.3	41

a Means and standard deviations represent data from three independent experiments.

b Reported previously in reference 20.

10.1128/mBio.01894-18.1 FIG S1 Microcalorimetric binding studies of McpU-LBD. (A) Titrations of 17.5 µM McpU-LBD with 1 mM histamine and ethylenediamine and 30 µM McpU-LBD with 0.5 mM agmatine. (B) Chemical structure of ligands recognized by the McpU and TlpQ chemoreceptors. Download FIG S1, JPG file, 0.6 MB. Copyright © 2018 Corral-Lugo et al. 2018 Corral-Lugo et al. This content is distributed under the terms of the Creative Commons Attribution 4.0 International license.

### Identification of TlpQ as a histamine receptor in Pseudomonas aeruginosa.

**FIG 2:**
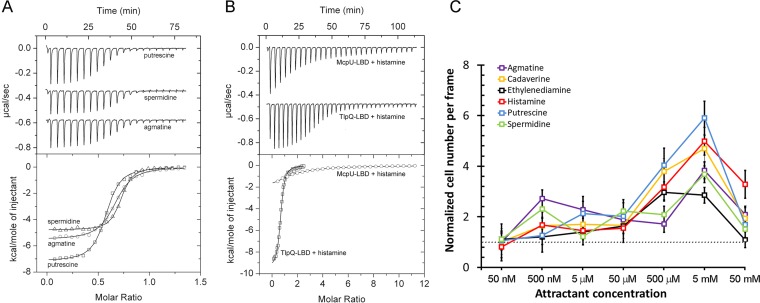
Identification and analysis of TlpQ ligands. (A) Microcalorimetric titrations of 15 µM TlpQ-LBD with 4.8 µl aliquots of 250 µM putrescine, spermidine, or cadaverine. (B) Microcalorimetric titration of 17.5 µM McpU-LBD with 9.6 µl aliquots of 1 mM histamine and titration of 15 µM TlpQ-LBD with 4.8 µl aliquots of 250 µM histamine. Upper graphs show raw titration data, while lower graphs show integrated corrected peak areas of the titration data fit using the “one binding site model.” The derived thermodynamic parameters are provided in Table 1. (C) Quantitative capillary chemotaxis assays of P. aeruginosa PAO1 toward TlpQ ligands. Shown are the ratios of cells after 2 min of exposure to the chemoattractant relative to the number of cells at the beginning of the experiment. The horizontal line marks the ratio of 1, which is indicative of no chemotaxis; *n* = 3.

Because histamine plays an important role in the immune response, we aimed to identify McpU homologues in P. aeruginosa that may also sense and mediate chemotaxis to histamine. To this end, we carried out a sequence clustering analysis of all dCACHE-containing chemoreceptors in PAO1 and KT2440 (see Fig. S2A). This analysis revealed that the LBD of the TlpQ receptor shares 62% sequence identity with the McpU-LBD homologue (Fig. S2B). To verify TlpQ function, we purified TlpQ-LBD for ITC binding studies. The results showed that five McpU-LBD ligands bind to TlpQ-LBD with nanomolar affinities, whereas the binding of ethylenediamine was slightly weaker (Fig. 2A, Table 1). Spermidine had a *K_D_* of 56 nM, which is the highest ligand affinity ever observed for a chemoreceptor. Histamine had a *K_D_* of 639 nM, which is an affinity 41 times higher than its affinity for McpU-LBD (Table 1, Fig. 2B). Thus, the affinities of the ligands to TlpQ-LBD were 3 to 147 times higher than their affinities to the McpU-LBD (Table 1). Previous studies showed that TlpQ mediates chemotaxis to ethylene (31), but the titration of TlpQ-LBD with a saturated ethylene solution did not show any binding (data not shown).

10.1128/mBio.01894-18.2 FIG S2 Identification of a McpU homologue. (A) Sequence clustering of the ligand binding domains of dCACHE-containing chemoreceptors from P. putida KT2440 (blue) and P. aeruginosa PAO1 (green). The figure was produced using the Phylogeny.fr server. (B) Sequence alignment of the TlpQ and McpU chemoreceptors. Sequence identity between the two receptors is 62%. The alignment was done in the slow mode using the CLUSTALW multiple alignment tool of the NPSA suite (https://npsa-prabi.ibcp.fr/cgi-bin/npsa_automat.pl?page=/NPSA/npsa_server.html). The GONNET protein weight matrix was used in the slow pairwise alignment mode using a gap opening penalty of 10 and a gap extension penalty of 0.1. Red, identical; green, highly similar; blue, weakly similar. The transmembrane regions flanking the ligand binding domain were predicted using the DAS algorithm (https://tmdas.bioinfo.se/DAS/index.html) and are highlighted in yellow. Cyan highlights amino acids that are involved in side-chain mediated interactions with bound histamine. Download FIG S2, TIF file, 1.1 MB. Copyright © 2018 Corral-Lugo et al. 2018 Corral-Lugo et al. This content is distributed under the terms of the Creative Commons Attribution 4.0 International license.

### Characterization of histamine chemotaxis.

**FIG 3:**
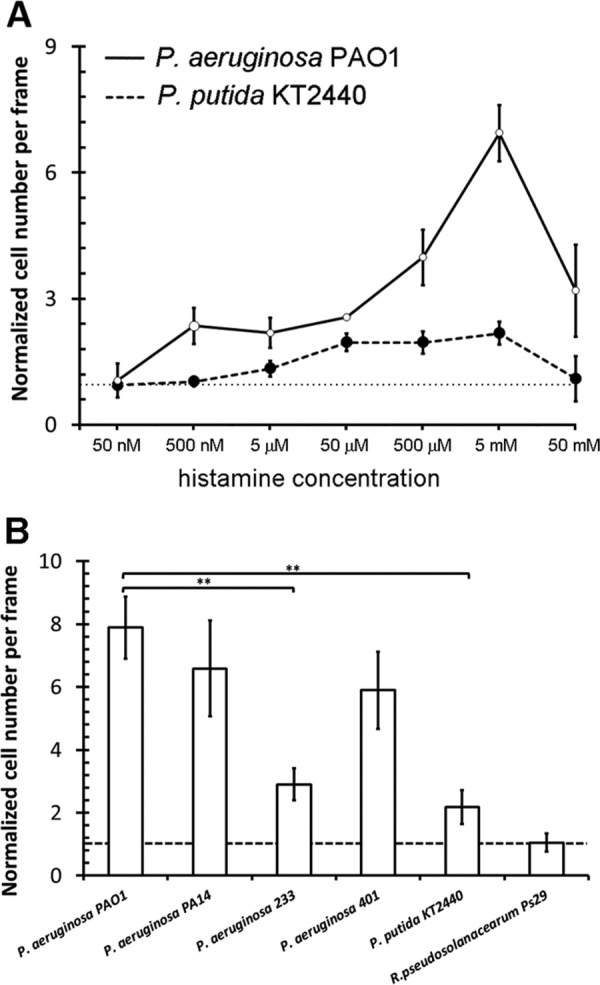
Histamine chemotaxis in different bacteria. (A) Quantitative capillary chemotaxis assays of P. aeruginosa PAO1 and P. putida KT2440 to different histamine concentrations. (B) Response of different strains to 5 mM histamine; *n* = 3. **, *P* < 0.01 (by Student’s *t* tests).

KT2440 and PAO1 both contain chemoreceptors that bind histamine. In initial experiments, we identified the optimal culture conditions for motility of both strains (see Fig. S3). Using these conditions, we carried out capillary chemotaxis assays of PAO1 toward the six TlpQ ligands (Fig. 2C). All ligands caused chemotaxis, with significant responses observed for some ligands at concentrations as low as 500 nM, whereas optimal responses occurred at 5 mM. In subsequent experiments, we compared the histamine dose response for KT2440 with that of PAO1 (Fig. 3A). KT2440 showed only moderate chemotaxis over the entire concentration range tested, whereas PAO1 responses were much stronger. In accordance with the different binding affinities observed by ITC, the response onsets between strains also differed. Thus, PAO1 required 500 nM histamine, while KT2440 required 5 µM.

10.1128/mBio.01894-18.3 FIG S3 Assessment of motility during bacterial growth. Overnight cultures of P. aeruginosa PAO1 (continuous line) and P. putida KT2440 (dotted line) were used to inoculate LB (upper panel) and 2× YT medium (lower panel) to an OD_600_ of 0.01. Growth was carried out at 37°C (P. aeruginosa PAO1) or 30°C (P. putida KT2440), and bacteria were inspected microscopically. Motility scores were calculated as follows: score 1, 25% of bacteria are motile; 2, 50%; 3, 75%; and 4, 100%. Download FIG S3, TIF file, 0.1 MB. Copyright © 2018 Corral-Lugo et al. 2018 Corral-Lugo et al. This content is distributed under the terms of the Creative Commons Attribution 4.0 International license.

To assess the metabolic value of these ligands, we conducted growth experiments with PAO1 and KT2440 in minimal medium containing each of the ligands as the sole carbon or nitrogen source. We found that most of the ligands supported growth either as the carbon or nitrogen source (see Fig. S4). The exceptions were spermidine and ethylenediamine that were either not or were poor growth substrates for PAO1 and KT2440 (Fig. S4). Histamine permitted the growth of both strains as the sole C and N source.

10.1128/mBio.01894-18.4 FIG S4 Growth experiments with McpU/TlpQ ligands as sole carbon or nitrogen source. (A) Capacity of ligands to sustain growth of P. aeruginosa PAO1 and P. putida KT2440. Overnight cultures were used to inoculate MS minimal medium supplemented with the McpU/TlpQ ligands as either sole carbon or nitrogen source. As controls, succinate and ammonium nitrate were used. (B) Growth of Ralstonia pseudosolanacearum Ps29 in medium with histamine as the sole carbon and nitrogen source. The initial OD_660_ was 0.005. The composition of the RSM medium was 10 mM K_2_HPO_3_, 5.5 mM KH_2_PO_4_, 0.5 mM sodium citrate, 9.5 mM (NH_4_)_2_SO_4_, 1 mM MgSO_4_, and 28 mM glucose. In all cases, the ligands were added at a concentration of 5 mM, and shown are means and standard deviations from three biological replicates conducted in triplicates. Download FIG S4, JPG file, 0.8 MB. Copyright © 2018 Corral-Lugo et al. 2018 Corral-Lugo et al. This content is distributed under the terms of the Creative Commons Attribution 4.0 International license.

Additional experiments were conducted to assess histamine chemotaxis in other bacteria. First, we assessed the motility of P. aeruginosa strains 227, 233, 287, 401, and 428, which were isolated from patients with urinary tract infections (41). Strains 233 and 401 exhibited motilities comparable to that of PAO1 and were therefore selected for further studies. P. aeruginosa PA14 as well as the plant pathogen Ralstonia pseudosolanacearum Ps29 were also included in these experiments. We found that all analyzed P. aeruginosa strains showed significant chemotaxis to 5 mM histamine, and their chemotactic phenotype was significantly higher than that of KT2440. On the other hand, the strain Ps29 was not attracted to histamine (Fig. 3B). Growth experiments with Ps29 in minimal medium containing histamine as the sole carbon and nitrogen source revealed no significant growth (Fig. S4B), suggesting a link between chemotaxis and the capacity to use histamine for growth.

### Three-dimensional structure of TlpQ-LBD in complex with histamine.

**FIG 4:**
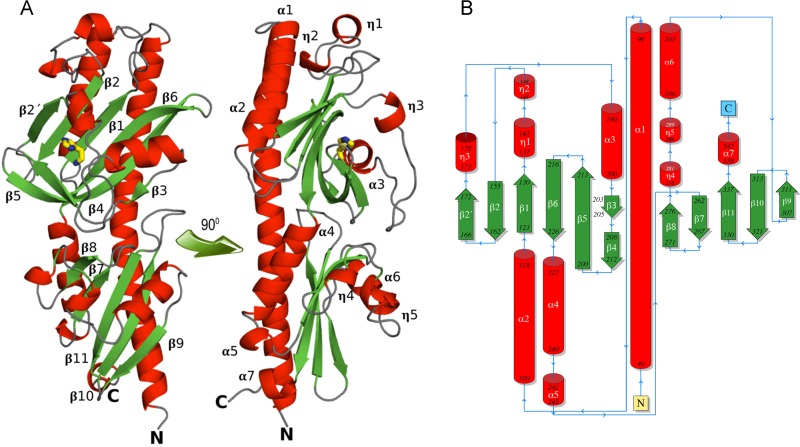
Structure of the TlpQ chemoreceptor ligand binding domain in complex with histamine. (A) Ribbon diagram with annotated secondary structure elements. Bound histamine is shown as a stick structure. (B) Schematic representation of the secondary structure elements.

To determine the molecular determinants for histamine recognition by TlpQ, we solved the high-resolution structure of TlpQ-LBD in complex with histamine. There are four monomers in the asymmetric unit,and the superimposition of their C_α_ atoms resulted in root mean square deviation (RMSD) values of 0.4 to 0.8 Å, indicative of high similarity. An inspection of the structure revealed that it is a dCACHE domain (12) (Fig. 4). A long N-terminal helix is followed by two globular α/β modules, termed membrane-proximal and membrane-distal modules. The membrane-distal module contained bound histamine in all four monomers of the asymmetric unit (Fig. 4).

Structural alignments of TlpQ-LBD with entries in the protein data bank identified structural homologues (see Table S1). Most of the homologues are categorized as dCACHE_1 Pfam domains (12). This domain is found in histidine kinases and chemoreceptors, as well as in a novel cytosolic receptor protein (PDB identifier [ID] 5ere), and are found in different bacteria as well as in *Arabidopsis*. The average size of the domains, while taking into account the segment in between both transmembrane regions, is 268 ± 17 amino acids (Table S1). Of all the homologous domains that we identified, TlpQ-LBD was the largest, at 334 amino acids, namely due to particularly long inserts between β-strands 1 and 2, extended helices α1 and α2, and an extended loop between helices η3 and α3 (see Fig. S5).

10.1128/mBio.01894-18.5 FIG S5 Structural alignment of the Cα chain of TlpQ-LBD (in red) with a homologous structure from a histidine kinase of Shewanella oneidensis (in green). This structure is deposited in the protein data bank with ID 3lic. The arrows indicate inserts and loops in the TlpQ-LBD structure that account for its elevated size. Download FIG S5, JPG file, 0.7 MB. Copyright © 2018 Corral-Lugo et al. 2018 Corral-Lugo et al. This content is distributed under the terms of the Creative Commons Attribution 4.0 International license.

10.1128/mBio.01894-18.8 TABLE S1 Structural alignment of TlpQ-LBD with structures deposited in the protein data bank. Shown are the structures with a Z-score above 15. Download Table S1, DOCX file, 0.03 MB. Copyright © 2018 Corral-Lugo et al. 2018 Corral-Lugo et al. This content is distributed under the terms of the Creative Commons Attribution 4.0 International license.

**FIG 5:**
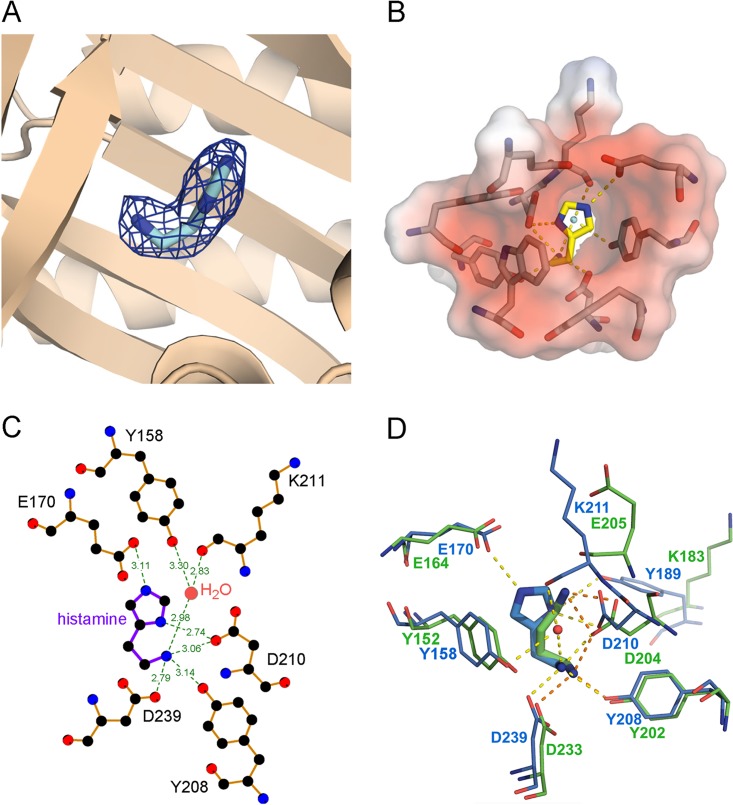
Ligand binding pocket of the TlpQ ligand binding domain. (A) Close-up view of the ligand binding pocket. The electron density for histamine is shown. (B) Surface charge representation of the histamine binding site; red and blue shading represent negative and positive charges, respectively. (C) Schematic representation of amino acids involved in hydrogen bonds with histamine. (D) Superimposition of the ligand binding pockets of McpU-LBD with bound putrescine (green, PDB ID 6F9G) and TlpQ-LBD with bound histamine (blue).

A well-defined electron density for histamine was observed in all four monomers, enabling the ligand placement to be determined (Fig. 5A). TlpQ ligands are present as protonated polycations at neutral pH, which explains why the ligand binding pocket is highly negatively charged (Fig. 5B). All three histamine nitrogen atoms establish hydrogen bonds (Fig. 5C). TlpQ ligands contain at least one primary amino group, and the primary amino group of histamine plays a central role in binding because it forms hydrogen bonds with the side chains of Tyr208, Asp210, and Asp239. In addition, this histamine amino group interacts with a water molecule coordinated by the main chain oxygen of Lys211 and the hydroxyl group of Tyr158. Each of the histamine imidazole nitrogen atoms forms hydrogen bonds with Asp210 and Glu170. The LBDs of TlpQ and McpU of P. putida KT2440 share approximately 50% sequence identity (Fig. S2B). When their structures containing either histamine or putrescine were superimposed (Fig. 5D), it became apparent that the primary amino groups of both ligands are coordinated in a similar manner via hydrogen bonds with Y208/D210/D239 of TlpQ-LBD or their equivalents in McpU-LBD.

### Histamine chemotaxis is mediated by multiple chemoreceptors in PAO1.

**FIG 6:**
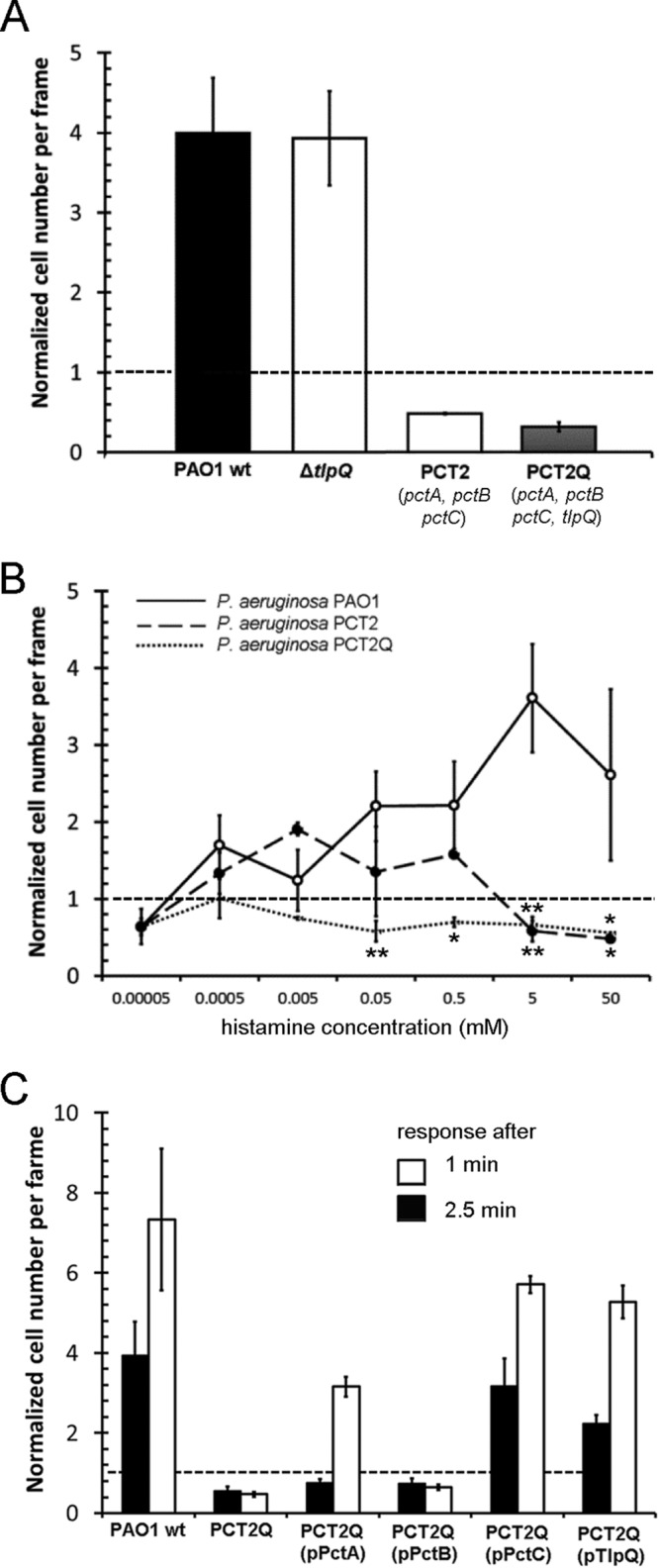
Chemotaxis to histamine is mediated by multiple chemoreceptors in P. aeruginosa PAO1. (A) Chemotactic responses to 5 mM histamine by wild-type and mutant strains. (B) Histamine dose-response chemotaxis assays for PAO1 and for PCT2 and PCT2Q mutants. (C) Chemotactic response of PAO1 and PCT2Q harboring plasmids pPctA, pPctB, pPctC, and pTlpQ to 500 µM histamine after contact times of 1 min and 2.5 min; *n* = 3. *, *P* < 0.05; **, *P* < 0.01 (by Student’s *t* tests).

To assess the role of TlpQ in histamine chemotaxis, we generated a *tlpQ* mutant. Control experiments showed that its response to Casamino Acids was comparable to that of the wild type. However, the response of this mutant to 5 mM histamine was also similar to that of the wild type (Fig. 6A), indicating that additional chemoreceptors may be involved.

To identify these additional chemoreceptors, we screened a number of mutants, in which 3 to 7 chemoreceptor genes had been deleted. Our results showed that the deletion of the *pctA*, *pctB*, and *pctC* chemoreceptor genes (strain PCT2) abolished chemotaxis to 5 mM histamine (Fig. 6A). PctA, PctB, and PctC are chemoreceptors for l-amino acids (23, 24), while PctC also mediates chemotaxis toward GABA (21).

To clarify the roles of PctA, PctB, PctC, and TlpQ in histamine chemotaxis, we conducted dose-response experiments using wild-type PCT2 as well as a mutant in which the *pctABC* as well as the *tlpQ* gene had been deleted, named PCT2Q (Fig. 6B). The latter mutant was devoid of histamine chemotaxis over the entire concentration range (50 nM to 50 mM), whereas significant chemotaxis was observed for the PCT2 mutant at a concentration range between 500 nM and 500 µM. This indicates that TlpQ mediates chemotaxis to low histamine concentrations, which is in agreement with the very high affinity observed *in vitro*. In contrast, one or more of the PctA, PctB, and PctC receptors mediate chemotaxis to elevated histamine concentrations.

To assess the role of the individual chemoreceptors, the PCT2Q mutant devoid of histamine chemotaxis was complemented with plasmids containing one of the four chemoreceptors—an approach that has previously proven effective to study complex chemotactic processes (42). To confirm the phenotypes of these strains, chemotaxis was measured toward previously identified ligands, namely l-Ile (PctA), l-Arg (PctB), and GABA (PctC) (23, 24), and the three complemented strains responded to these ligands. Histamine chemotaxis measurements revealed that the *pctC* and *tlpQ* genes in *trans* recovered histamine chemotaxis using an exposure time of 1 min. At 2.5 min, complementation with *pctA*, *pctC*, and *tlpQ* resulted in significant chemotaxis (Fig. 6C). Thus, these data reveal that histamine chemotaxis is mediated by the concerted action of PctA, PctC, and TlpQ. To assess the role of these receptors in another strain, we generated a triple mutant in the homologous receptors of P. aeruginosa PA14. As shown in Fig. S6, the deletion of these receptors also abolished histamine chemotaxis.

10.1128/mBio.01894-18.6 FIG S6 Quantitative capillary chemotaxis assays of P. aeruginosa PA14 and a mutant defective in the *pctA*, *pctC*, and *tlpQ* genes towards histamine. Data have been corrected with the number of bacteria (6,900 ± 142) that swam into buffer-containing capillaries. Shown are means and standard deviations from three individual experiments conducted in duplicates. Download FIG S6, TIF file, 1.5 MB. Copyright © 2018 Corral-Lugo et al. 2018 Corral-Lugo et al. This content is distributed under the terms of the Creative Commons Attribution 4.0 International license.

### TlpQ, PctA, and PctC employ different mechanisms to mediate histamine chemotaxis.

To determine the mechanism by which PctA and PctC respond to histamine, microcalorimetric binding studies with purified PctA-LBD and PctC-LBD were conducted. Whereas the proteins bound l-Ala and l-Gln (24), respectively, histamine did not bind. Direct microcalorimetric titrations can only provide information on high-affinity binding events because of the limitations presented by ligand dilution heats. To assess the possibility of low-affinity histamine binding, we conducted a competition assay. PctA-LBD was titrated with l-Ala in the presence and absence of 20 mM histamine. However, the resulting titration curves were almost identical (see Fig. S7), confirming that histamine does not bind directly to PctA-LBD.

10.1128/mBio.01894-18.7 FIG S7 Assessment of potential low-affinity binding of histamine to PctA-LBD by microcalorimetric competition experiments. Shown are microcalorimetric titrations of 39 µM PctA-LBD with 1 mM l-alanine either in the absence or presence of 20 mM histamine. The resulting integrated peak areas are almost identical indicating that histamine does not compete with l-alanine for binding at PctA-LBD. Download FIG S7, JPG file, 0.2 MB. Copyright © 2018 Corral-Lugo et al. 2018 Corral-Lugo et al. This content is distributed under the terms of the Creative Commons Attribution 4.0 International license.

To assess whether PctA and PctC may be activated by histamine-containing periplasmic binding proteins, pulldown experiments with immobilized PctA-LBD and PctC-LBD as well as PAO1 protein extracts were conducted using previously verified protocols (25). However, our results provided no evidence for binding partners to either domain.

## DISCUSSION

The elevated numbers of chemoreceptors in many bacteria suggest that this abundance confers chemotactic capabilities to many different stimuli, and the scientific community is only beginning to explore the diversity of these responses. In general, chemoeffectors can be classified into three groups according to their physiological role. First, the majority of chemoattractants are important nutritional sources, as evidenced by numerous receptors that respond to different organic or amino acids (11). Second, chemoattraction has been observed for signal molecules such as plant hormones (31, 43), neurotransmitters (44), and quorum sensing signals (45), which inform bacteria about their environment. Lastly, chemoreceptors can signal the presence of compounds, such as histamine, that are involved in multiple functions.

Thus, what is the physiological relevance of chemotaxis toward histamine? One possibility is certainly that, like most of the other McpU/TlpQ ligands, histamine supports growth as the sole C and N source. However, chemotaxis to host signals has been shown for many different pathogens to be essential for efficient infection and virulence (46). Importantly, P. aeruginosa PAO1 was shown to greatly increase neutrophil histamine content and secretion in mouse models (36), and chemotaxis to this host-derived signal will result in an accumulation of bacterial cells at the infection site. This increase in bacterial cell density likely alters the expression of quorum-sensing-controlled genes, including those responsible for the production of virulence determinants and biofilm formation in P. aeruginosa (47). Nonetheless, the precise assessment of the role of histamine chemotaxis in the virulence of P. aeruginosa is technically a difficult undertaking, since it is unfeasible to generate a mutant that is deficient in histamine chemotaxis without impairing taxis to the remaining identified ligands for PctA (17 amino acids), PctC (GABA and 2 amino acids), and TlpQ (5 polyamines) (24).

The interference with motility and chemotaxis is an alternative strategy to block bacterial pathogens (48). Previous work has shown that some chemoreceptors recognize chemoattractants and antagonists (27, 49, 50), and the identification of antagonists that specifically interfere with histamine chemotaxis may thus be an alternative approach to modulate the virulence properties of P. aeruginosa. Remarkably, the identification of these antagonists may be facilitated by the resolution of the three-dimensional structure of TlpQ-LBD in complex with histamine (Fig. 4 and 5).

High sensitivity histamine responses are mediated by the TlpQ chemoreceptor, which binds histamine directly. TlpQ is in many aspects an atypical chemoreceptor. First, its LBD, which is 334 amino acids, is larger than any other known chemoreceptor LBD (11). Second, it has the highest affinity ever observed for the binding of a chemoattractant to the recombinant LBD of a chemoreceptor. Histamine binding occurred with an affinity of 639 nM, which is among the highest affinities observed for chemoattractants. This unusually high affinity permits responses to very low histamine concentrations, and the onset of chemotactic response occurred at the unusually low concentration of 500 nM (Fig. 2C).

Frequently, the deletion of a chemoreceptor abolishes taxis to a given compound, indicating that there is a single receptor for a given chemoattractant (18, 29). However, histamine chemotaxis is mediated by the concerted action of three receptors. Thus, what is the advantage of having multiple receptors for the same chemoattractant? In this context, close similarities exist between histamine chemotaxis and the mechanisms by which P. aeruginosa is attracted to inorganic phosphate (P_i_). P_i_ is a key signaling molecule that controls the expression of many virulence genes (51, 52). Chemotaxis to a low P_i_ concentration is mediated by the CtpL receptor, whereas CtpH is responsible for responses to high concentrations (25, 26). Whereas CtpH recognizes P_i_ directly at its LBD, CtpL is stimulated by the P_i_-loaded periplasmic binding protein PstS (25). A chemoreceptor, either stimulated by direct or indirect signal recognition, is characterized by a response range (53). The combined action of multiple chemoreceptors with different sensing abilities permits the microorganism to expand its response range to a given chemoattractant. The presence of multiple receptors for a given chemoeffector may suggest that a compound is particularly physiologically relevant.

Four human histamine receptors have been described, termed H1, H2, H3, and H4 (54). Histamine was shown to mediate the chemotaxis of mast cells via the H4 receptor, and this mechanism might be responsible for mast cell accumulation in allergic tissues (55). However, the topology of eukaryotic histamine receptors differs entirely from that of their bacterial counterparts. All four receptor types form a barrel composed of seven transmembrane helices (54). The three-dimensional structure of the human H1 receptor has been solved (56), which revealed that ligands bind within this transmembrane barrel. Therefore, the evolutionary strategies to sense histamine greatly differ between bacteria and humans.

Here, we provide the first report of bacterial chemotaxis toward histamine. Histamine is vital to cellular processes in mammals, and the initial evidence suggests that histamine also functions as a bacterial signal molecule. This study expands the range of known bacterial chemoeffectors and lays the foundation for deciphering the molecular mechanisms underlying histamine chemotaxis and its role in bacterial virulence.

## MATERIALS AND METHODS

### Bacterial strains and plasmids.

The bacterial strains and plasmids used are listed in Table 2, and oligonucleotides are listed in Table S2 in the supplemental material.

**TABLE 2:** Bacterial strains and plasmids used in this study

Strain or plasmid	Characteristics^*1*^	Reference or source
Strains		
Escherichia coli BL21(DE3)	F^−^ *ompI hsdS*_B_(r_B_^−^ m_B_^−^) *gal dam met*	69
DH5α	*supE44 lacU169*(φ80*lacZ*ΔM15) *hsdR17* (r_K_^−^ m_K_^−^) *recA1 endA1 gyrA96 thi-1 relA1*	70
HB101	F^−^ Δ(*gpt-proA*)*62 leuB6 supE44 ara-14 galK2 lacY1* Δ(*mcrC-mrr*) *rpsL20* (Sm^r^) *xyl-5 mtl-1 recA13 thi-1*	71
JM109	F′ *traD36 proA^+^B^+^ lacI*^q^ Δ(*lacZ*)M15 Δ(*lac-proAB*) *glnV44 e14*^−^ *gyrA96 recA1 relA1 endA1 thi hsdR17*	72
S17-1 λ*pir*	Tp^r^ Sm^r^ (*rec*A *thi pro hsdR*)^−^ M*+RP4*: 2-Tc:Mu: Km Tn*7* λ*pir*	73
Ralstonia pseudosolanacearum Ps29	Wild-type strain race 1, biovar 3, phylotype I	74
Pseudomonas putida KT2440	Wild type	15
KT2440R	Rifampicin-resistant derivative of KT2440	75
KT2440R-McpU	KT2440R transposon mutant *pp1228*::mini-Tn*5*-Km; Rif^r^, Km^r^	76
Pseudomonas aeruginosa PAO1	Wild-type strain	77
PAO1 Δ*pctA*	PAO1 derivative, *pctA* gene deletion mutant	This study
PCTB1	PAO1 derivative, *pctB*::Km; Km^-^	23
PCTC1	PAO1 derivative, *pctC*::Km; Km^-^	23
PAO1 Δ*tlpQ*	PAO1 derivative, *pa2654* gene deletion mutant	This study
PCT2	PAO1 derivative; Δ*pctB-pctA*-*pa4308*-*pctC*::km; Km^r^^*b*^	23
PCTAQ	PAO1 derivative; Δ*pctA* Δ*tlpQ*	This study
PCT2Q	PAO1 derivative; Δ*pctB-pctA*-*pa4308*-*pctC*::Km, Δ*tlpQ*; Km^r^	This study
PCT2QP	PAO1 derivative; Δ*pctB*-*pctA*-*pa4308*-*pctC*::Km, Δ*tlpQ* Δ*tlpP*; Km^r^	J. Kato lab
PCT2QART	PAO1 derivative; Δ*pctB-pctA*-*pa4308*-*pctC*, Δ*tlpQ* Δ*tlpA* (*pa1646*), Δ*tlpR*(*pa2652*) Δ*tlpT*(*pa1930*); Km^r^	J. Kato lab
P. aeruginosa PA14	Wild-type strain; human clinical isolate that elicits disease in plants, nematodes, insects, and mice	78
PA14-ACQ	PA14 derivative; Δ*pctA* Δ*pctC* Δ*tlpQ* Δ*pa4308*	This study
P. aeruginosa isolate 227	Isolated clinical strain from patients with urinary tract infections	41
P. aeruginosa isolate 233	Isolated clinical strain from patients with urinary tract infections	41
P. aeruginosa isolate 287	Isolated clinical strain from patients with urinary tract infections	41
P. aeruginosa isolate 401	Isolated clinical strain from patients with urinary tract infections	41
P. aeruginosa isolate 428	Isolated clinical strain from patients with urinary tract infections	41
Plasmids		
pUCP18	*Escherichia*-*Pseudomonas* shuttle vector; Ap^r^	79
pPctA	pUCP18 with a PCR fragment containing *pctA*; Ap^r^	80
pPctB	pUCP18 with a PCR fragment containing *pctB*; Ap^r^	80
pPctC	pUCP18 with a PCR fragment containing *pctC*; Ap^r^	This study
pTlpQ	pUCP18 with a PCR fragment containing *tlpQ*; Ap^r^	27
pK18*mobsacB*	Plasmid for allelic exchange; pK18 *oriV_E.c._lacZα mob sacB*; Km^r^	81
pK18*mobsacB-pctA*	pK18*mobsacB* containing a deletion of the *pctA* gene; Km^r^	This study
pK18*mobsacB-tlpQ*	pK18*mobsacB* containing a deletion of the *tlpQ* gene; Km^r^	This study
pK18*mobsacB*-*pctABC*	pK18*mobsacB* containing a deletion of *pctB*, *pctA*, *pa4308*, *pctC*; Km^r^	This study
pK18*mobsacB-pctC*	pK18*mobsacB* containing a deletion of *pctC* and *pa4308*; Km^r^	This study
pET28b(+)	Protein expression plasmid; Km^r^	Novagen
pET28b-McpU	pET28b derivative used to produce His-tagged McpU-LBD; Km^r^	20
pET28b-TlpQ	pET28b derivative used to produce His-tagged TlpQ-LBD; Km^r^	This study

a Ap, ampicillin; Km, kanamycin; Rif, rifampin.

b The *pa4308* gene (orf-1), which forms part of the *pctABC* operon, encodes a hypothetical protein that is not involved in chemotaxis (23).

10.1128/mBio.01894-18.9 TABLE S2 Oligonucleotides used in this study. Download Table S2, DOCX file, 0.02 MB. Copyright © 2018 Corral-Lugo et al. 2018 Corral-Lugo et al. This content is distributed under the terms of the Creative Commons Attribution 4.0 International license.

### Construction of bacterial mutant strains and plasmids.

The *pctA* and *tlpQ* genes were deleted in different mutant strains by unmarked gene deletion. Plasmids pK18*mobsacB*-*pctA* and pK18*mobsacB*-*tlpQ* were generated by amplifying 0.6- to 1.2-kb regions up- and downstream of the target gene. PCR products were digested with the restriction enzymes listed in Table S2 and cloned into pK18*mobsacB*. The resulting plasmids were introduced into E. coli S17-1 λ*pir* by electroporation. Plasmids were transferred to PAO1 by conjugation, and cells were selected in Simmons citrate (BBL; Becton, Dickinson) agar plates supplemented with kanamycin. For plasmid excision, LB medium was inoculated with a kanamycin-resistant colony and grown for 12 h, and then spread on LB plates containing 20% (wt/vol) sucrose. To construct the triple deletion mutant in *pctA*, *pctC*, and *tlpQ* in P. aeruginosa PA14, pK18*mobsacB*-*pctA* and pK18*mobsacB*-*tlpQ* were consecutively conjugated into PA14 to generate the double mutant. Subsequently, the plasmid pK18*mobsacB-pctC*, generated by amplifying regions up- and downstream of *pctC*, was conjugated into PA14 Δ*pctA* Δ*tlpQ.* Kanamycin-resistant colonies were grown on LB agar plates supplemented with 20% (wt/vol) sucrose for selection. For complementation purposes, the *pctC* gene was amplified by PCR and cloned into plasmid pUCP18 using the restriction enzymes listed in Table S2. The ligation mixture was electroporated into E. coli JM109, and transformants were selected on carbenicillin-containing LB plates. The resulting plasmid pPctC was transferred to PCTC1 and PCT2Q by electroporation.

### Construction of the TlpQ-LBD expression plasmid.

The DNA fragment encoding the LBD of TlpQ was amplified, digested with NdeI and BamHI, and cloned into pET28b(+) linearized with the same enzymes.

### Overexpression and purification of proteins.

PctA-LBD and PctC-LBD were overexpressed and purified as described in reference 24. McpU-LBD and TlpQ-LBD were generated as reported in reference 20.

### Thermal shift assay.

Thermal shift experiments were conducted as reported in reference in 57. McpU-LBD in polybuffer {5 mM Tris, 5 mM PIPES [piperazine-*N*,*N*′-bis(2-ethanesulfonic acid)], 5 mM MES [morpholineethanesulfonic acid], 10% glycerol [vol/vol], 150 mM NaCl, pH 7.0} was used at a final concentration of 10 µM. Biolog (Hayward, CA, USA) compound arrays PM3B (nitrogen sources), PM4A (phosphorous and sulfur sources), and PM5 (nutrient supplements) were used for screening. The compositions of these arrays are provided at http://208.106.130.253/pdf/pm_lit/PM1-PM10.pdf.

### Isothermal titration calorimetry.

Titrations were carried out in a VP microcalorimeter (MicroCal, Northampton, MA, USA) at 25°C. Proteins dialyzed into polybuffer were titrated with ligands in dialysis buffer. Typically, 15 to 30 µM protein was titrated with 0.25 to 1 mM ligand solutions. For ethylene binding studies, TlpQ-LBD was titrated with 12-µl aliquots of a saturated ethylene solution in polybuffer, prepared as reported in reference 31. The mean enthalpies from the injection of ligands into the buffer were subtracted from titration data prior to data fitting using the “one binding site model” of ORIGIN.

### Chemotaxis assays. (i) Soft agar plate assays.

Strains were grown overnight in M9 minimal medium containing 0.1% glucose (wt/vol), diluted to an optical density at 600 nm (OD_660_) of 1 with fresh medium, and washed twice with M9 medium. The pellet was resuspended in 1 ml M9 medium. Ten-microliter aliquots of 5 mM chemoattractant solutions were placed onto plates containing M9 medium, 2.5 mM glucose, and 0.25% (wt/vol) agar. Two-microliter aliquots of bacterial suspensions were placed horizontally to each of the chemoattractant spots. The plates were incubated at 30°C for 16 to 20 h.

### (ii) Quantitative capillary chemotaxis assays.

Two protocols were used that differed in the ways the cells were counted. The first protocol was used to generate the data shown Fig. S6, whereas the second protocol was used for the remaining chemotaxis experiments. In the first protocol, overnight cultures of strains were diluted to an OD_660_ of 0.05 in MS medium (58) supplemented with 6 mg·liter^−1^ Fe citrate, trace elements, and 15 mM glucose and grown at 37°C. At an OD_660_ of 0.4, the cultures were centrifuged at 1,700 × *g* for 5 min and the pellet was washed twice with chemotaxis buffer (50 mM potassium phosphate, 20 mM EDTA, 0.05% [vol/vol] glycerol, pH 7.0). The cells were resuspended in this buffer and adjusted to an OD_660_ of 0.1, and 230-µl aliquots were placed into 96-well plates. Capillary tubes (P1424, Microcaps; Drummond Scientific) were heat sealed at one end and filled with chemotaxis buffer or chemotaxis chemoattractant solution. The capillaries were then immersed in bacterial suspensions at their open ends. After 30 min at room temperature, the capillaries were removed and rinsed with sterile water, and the content was expelled into 1 ml of M9 medium. Serial dilutions were plated on LB medium, and the CFU were determined. In all cases, the data were corrected to the number of cells that swam into the buffer-containing capillaries. In the second protocol, we used computer-assisted image analysis as reported previously (59). Briefly, capillaries were filled with chemoattractant solutions in 10 mM HEPES buffer (pH 7.0) containing 1% (wt/vol) agarose and heat sealed on one side. The cells were grown in 2× yeast extract-tryptone (YT), medium and 10-μl aliquots of the cell suspension were placed onto a microscope slide within the U-shaped spacer, which was then covered by a coverslip. The chemoattractant-filled capillaries were introduced into the chemotaxis chamber, and cell movement was videotaped, with images taken at the beginning and at different time intervals. If not otherwise stated, the contact time between the cell and the chemoattractant was 2 min. The Bioinformatics Assistant Icy Sport detector software (60) was used to determine the number of cells per image. The magnitude of chemotaxis was expressed as the number of cells after a given time over the number of cells at the beginning of the experiment. The data shown are the means and standard deviations from three experiments conducted in triplicates.

### Growth experiments.

PAO1 was grown overnight in MS minimal medium (29) containing 20 mM d-glucose. Cultures were diluted to an OD_600_ of 0.02 in MS medium supplemented with 5 mM carbon or nitrogen source. The assays were performed in 100-well polystyrene plates and incubated at 30°C (KT2440) or 37°C (PAO1) in a Bioscreen microbiological growth analyzer. The data represent the means and standard deviations from three biological replicates conducted in triplicates.

### Assessment of motility.

To assess bacterial motility, PAO1 and KT2440 were used to inoculate LB and 2× YT medium to an OD_600_ of 0.01. Growth was carried out at 37°C (PAO1) or 30°C (KT2440), and the bacteria were inspected microscopically. According to their motility, they were given different scores: score 1, 25% of bacteria are motile; 2, 50%; 3, 75%; and 4, 100%.

### Crystallization and structure resolution.

Crystallization trials were carried out with TlpQ-LBD in the absence and presence of histamine. TlpQ-LBD in polybuffer was incubated with a 2-fold molar excess of histamine on ice for 30 min. Unbound ligand was removed by buffer exchange using 10-kDa-cutoff filters (Amicon) and polybuffer. The apo- (6 mg/ml) and ligand-bound (26 mg/ml) proteins were loaded into 0.3-mm-diameter capillaries for counter diffusion crystallization using screen kits from Triana S & T (Granada, Spain). Only the LBD-TlpQ–histamine complex produced crystals in 1.5 M ammonium phosphate and 0.1 M sodium citrate pH 5.6. The same protocol was used to crystallize the Se-Met TlpQ-LBD. The capillaries were emptied into mother solution containing 10% to 25% (vol/vol) glycerol as the cryoprotectant. Crystals were diffracted at the European Synchrotron Radiation Facility and the Spanish Synchrotron ALBA. The data were indexed and integrated with XDS (61) and scaled with SCALA (62). All attempts to obtain a molecular replacement solution failed. Phases were obtained from the Se-methionine derivative by combining SAD data, at the selenium peak, with an initial model generated by Phyre2 (63), as input files for Auto-Rickshaw (64). All 26 expected heavy atom positions were identified by SHELXD (65) using the data to 3.5 Å. The model generated was refined with phenix.refine (66). Further refinement was performed against the best data set (2.45 Å) with phenix.refine (66) using Coot (67). Model quality was checked using MolProbity (68). The refinement statistics and quality indicators of the final model are summarized in Table S3. The structure was deposited at the protein data bank with identifier (ID) 6fu4.

10.1128/mBio.01894-18.10 TABLE S3 Data collection and refinement statistics of the three-dimensional structure of TlpQ-LBD (values in parentheses are for highest resolution shell). Download Table S3, DOCX file, 0.01 MB. Copyright © 2018 Corral-Lugo et al. 2018 Corral-Lugo et al. This content is distributed under the terms of the Creative Commons Attribution 4.0 International license.
