# Supplementary figures and images for: High-Affinity Chemotaxis to Histamine Mediated by the TlpQ Chemoreceptor of the Human Pathogen Pseudomonas aeruginosa
Source: mBio. 2018 Nov 13;9(6):e01894-18. doi: 10.1128/mBio.01894-18 (PMC6234866; doi:10.1128/mBio.01894-18)

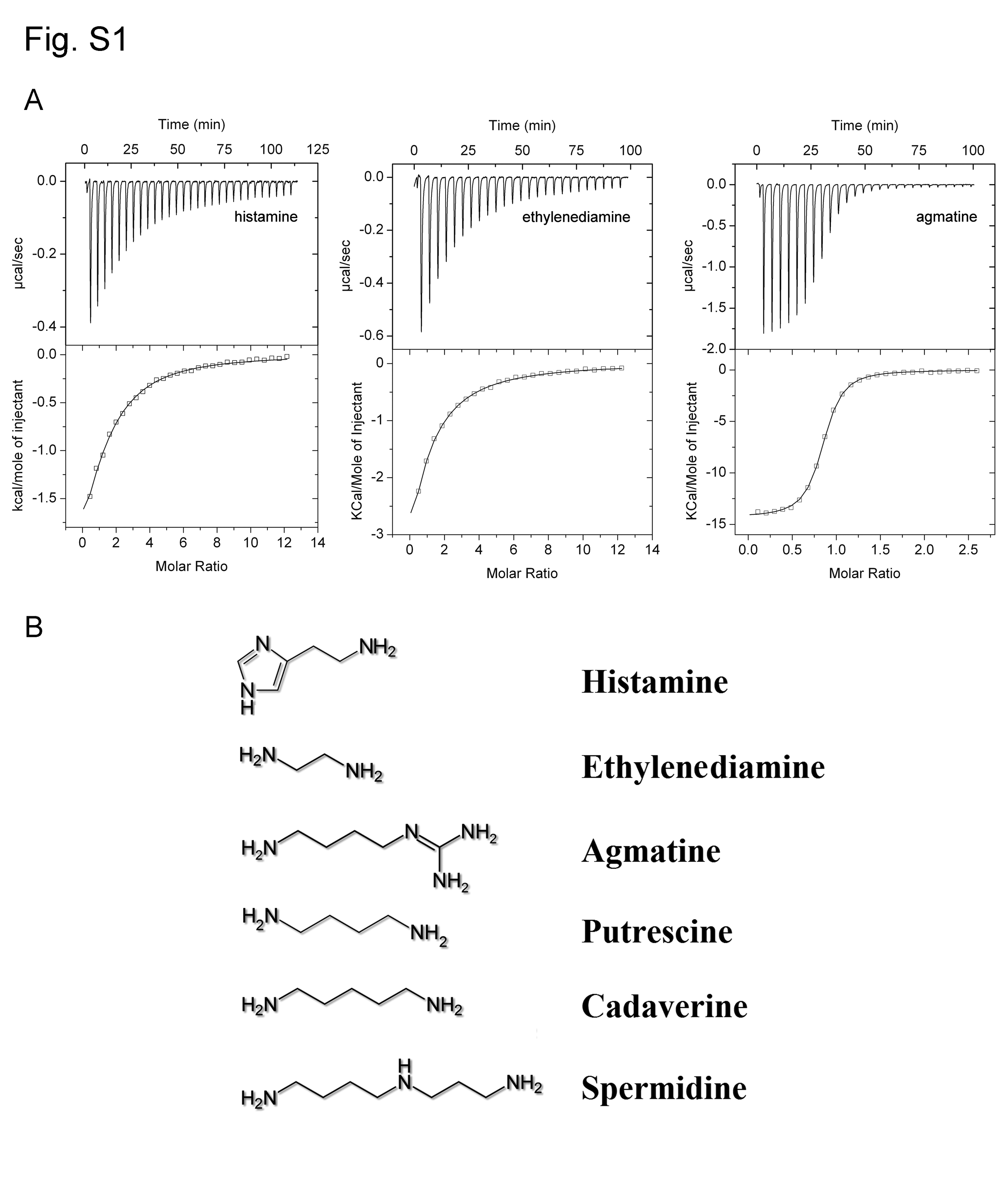

Supplement: FIG S1 [file mbo006184178sf1.jpg]

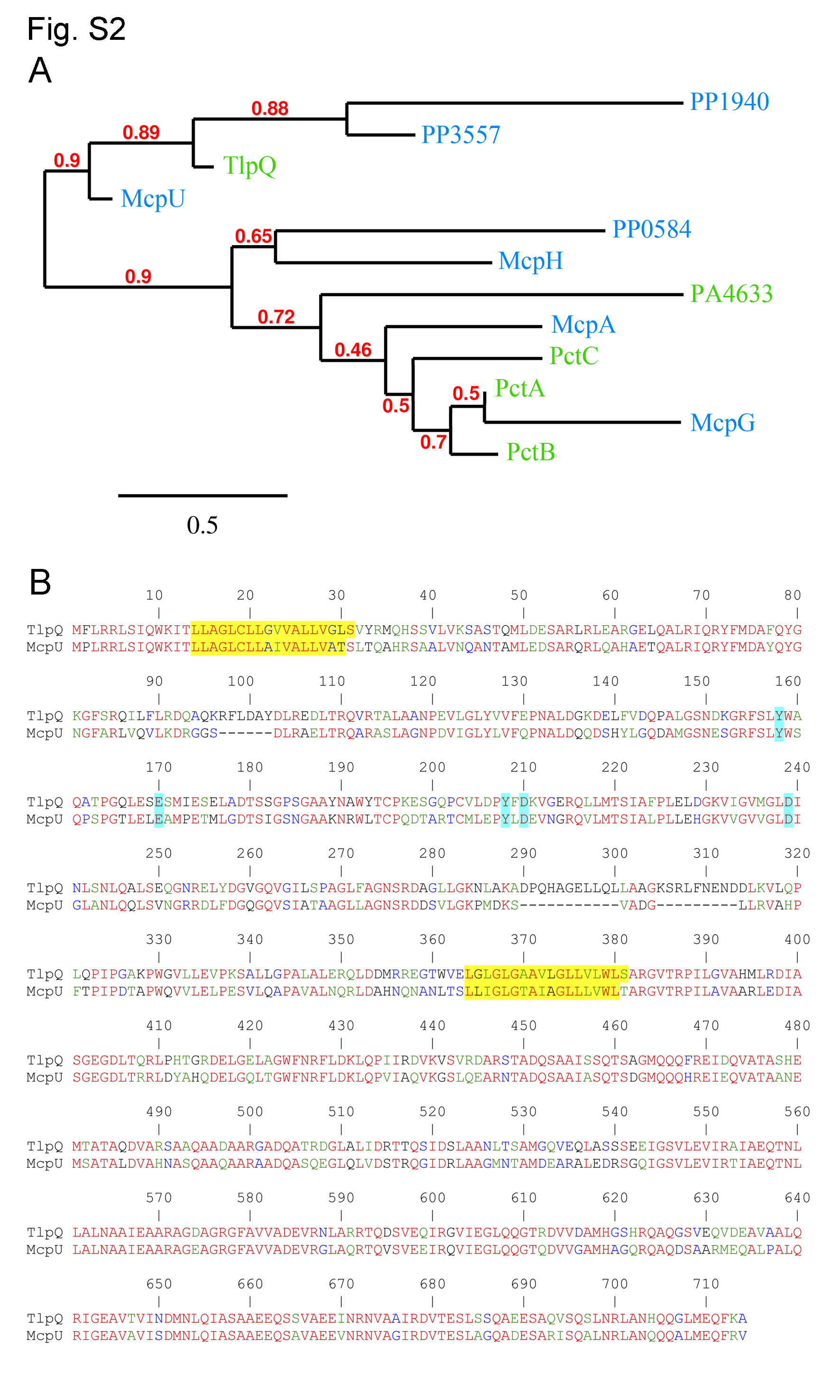

Supplement: FIG S2 [file mbo006184178sf2.tif]

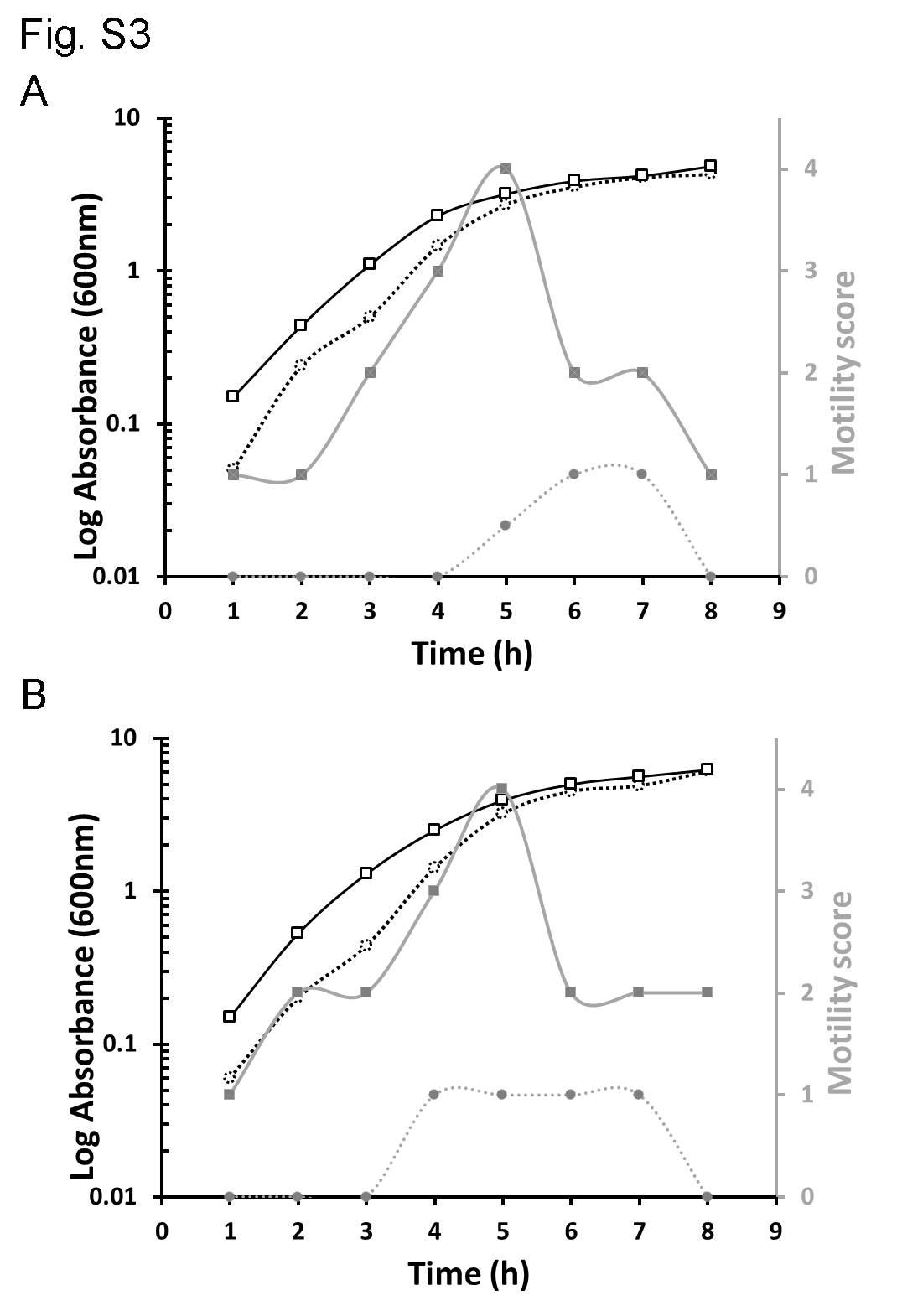

Supplement: FIG S3 [file mbo006184178sf3.tif]

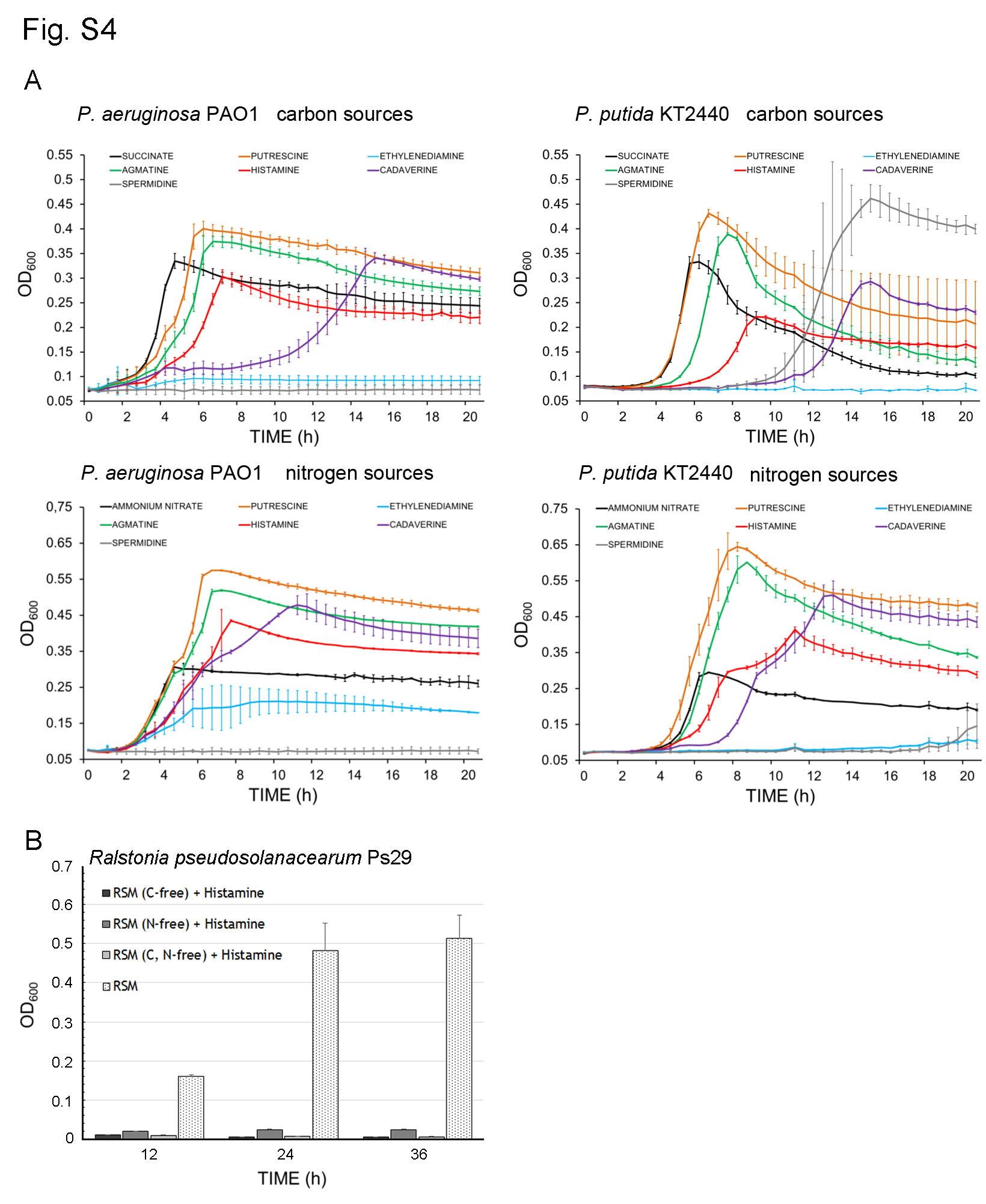

Supplement: FIG S4 [file mbo006184178sf4.jpg]

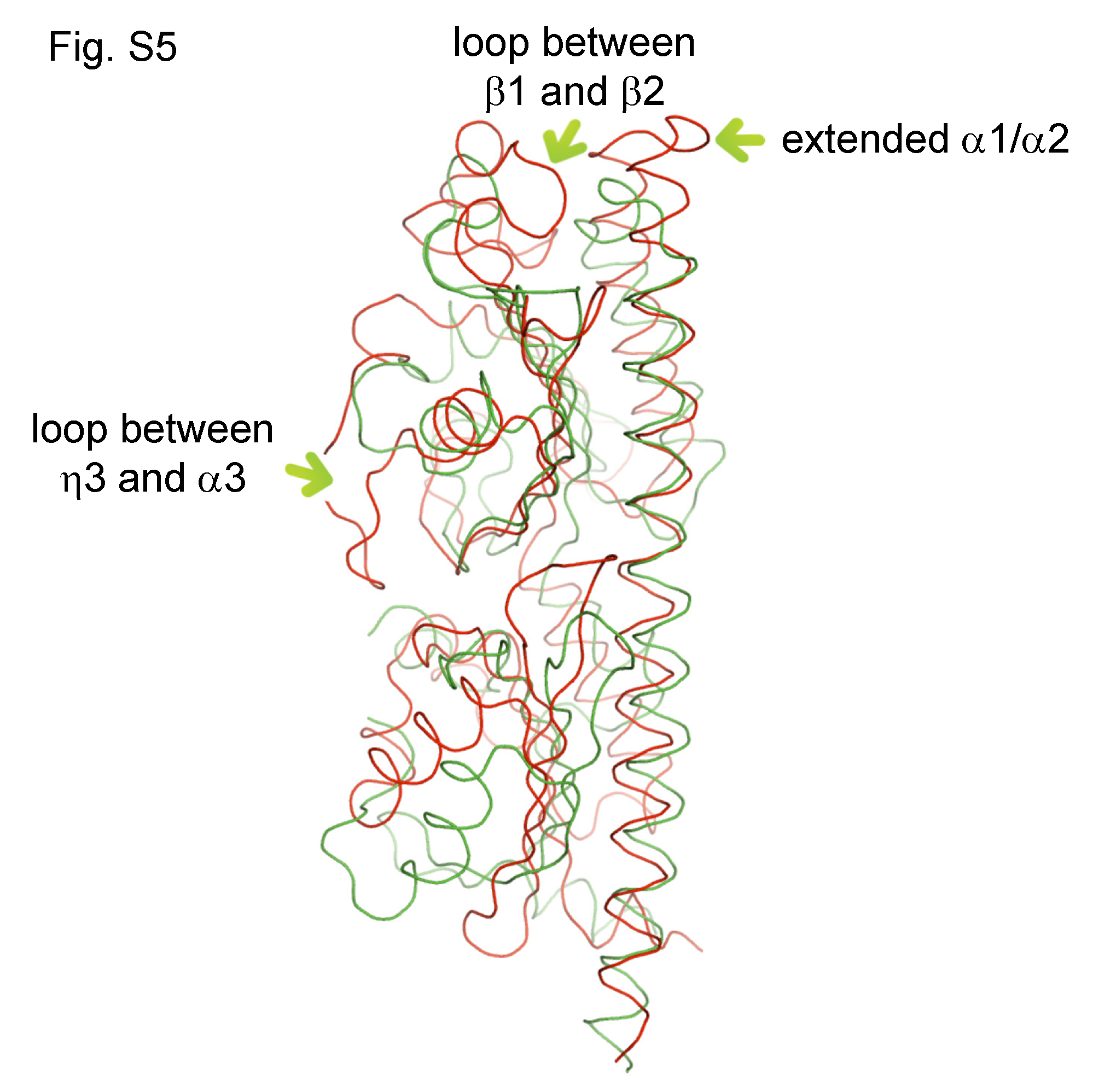

Supplement: FIG S5 [file mbo006184178sf5.jpg]

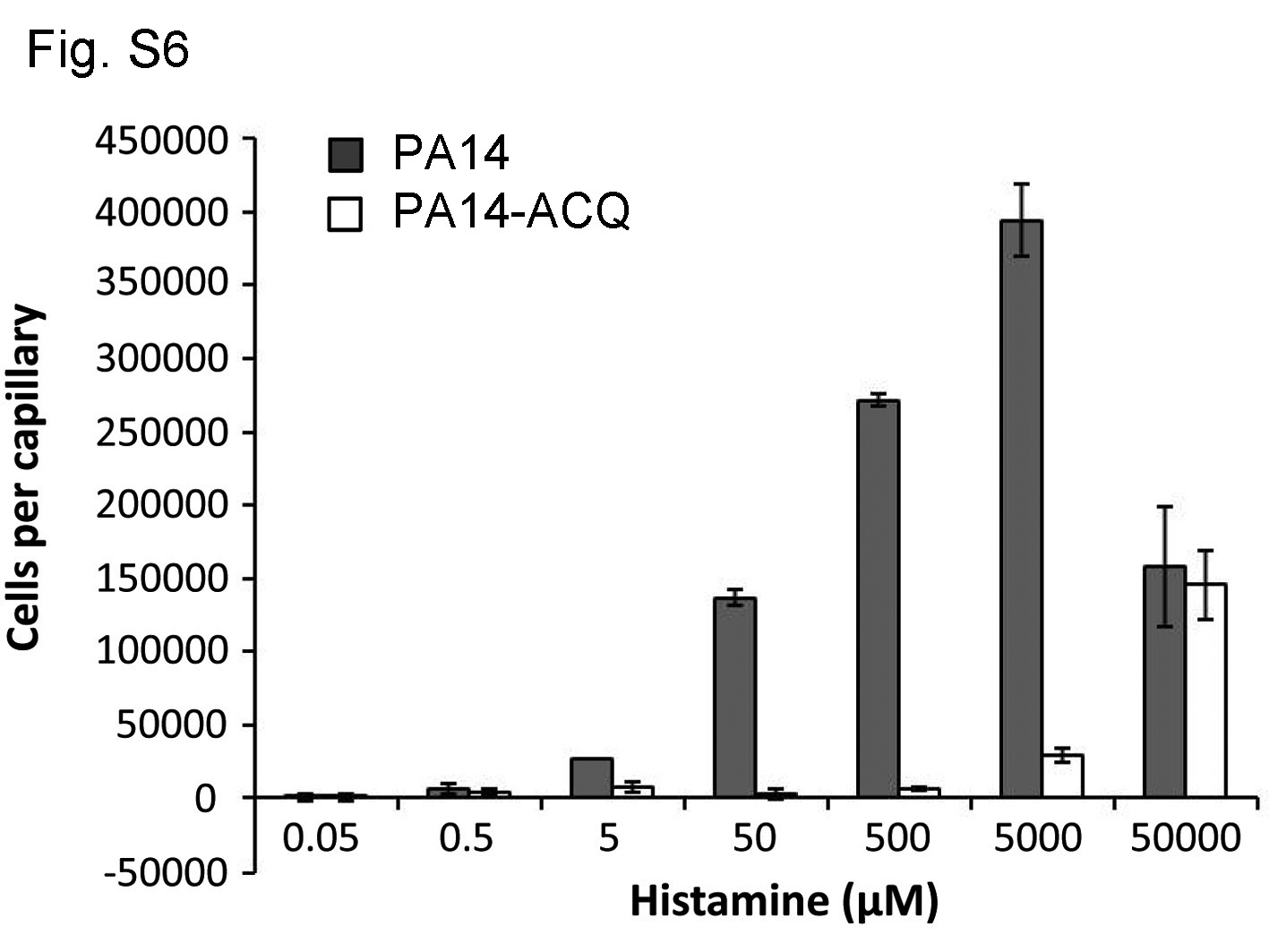

Supplement: FIG S6 [file mbo006184178sf6.tif]

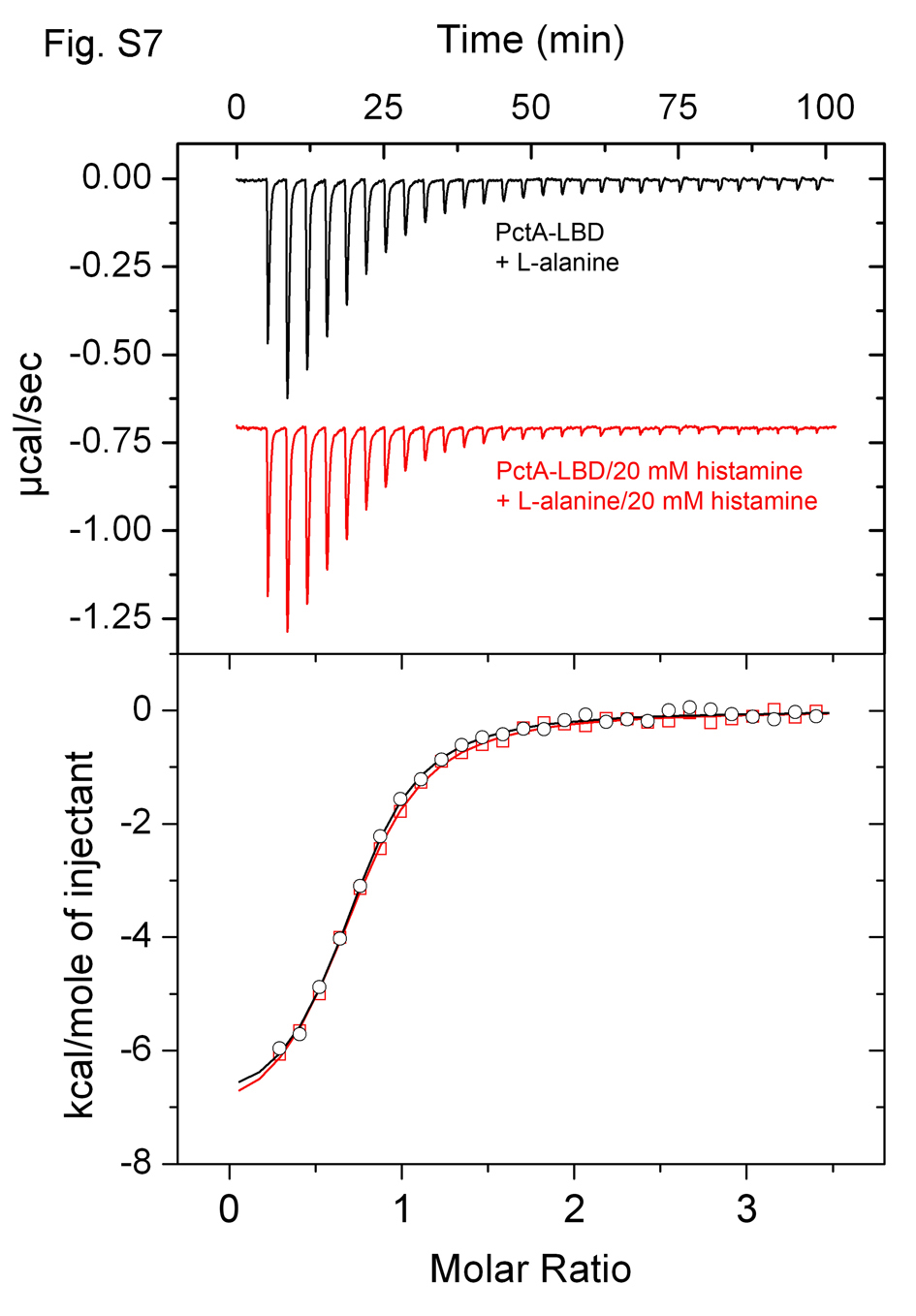

Supplement: FIG S7 [file mbo006184178sf7.jpg]
