# Supplementary material for: High-Affinity Chemotaxis to Histamine Mediated by the TlpQ Chemoreceptor of the Human Pathogen Pseudomonas aeruginosa
Source: mBio. 2018 Nov 13;9(6):e01894-18. doi: 10.1128/mBio.01894-18 (PMC6234866; doi:10.1128/mBio.01894-18)
Supplement: TABLE S1 [file mbo006184178st1.docx]

**Table S1.**

| PDB ID | name | Z-score | Identity  (%) | RMSD | LBD size^a^ | Ligands | Protein/species | Loop between β1-β2 (size) | Extended α1/α2 (size) | Loop between η3-α3 (size) | Pfam  signature | Ref. |
| --- | --- | --- | --- | --- | --- | --- | --- | --- | --- | --- | --- | --- |
| 6fu4 | TlpQ | - | - | - | 334 | histamine, polyamines | CR^b^/*P. aeruginosa* | Yes (22)^c^ | Yes (5/4)^c^ | Yes (9)^c^ | dCACHE_1 | This work |
| 6f9g | McpU | 37.1 | 56 | 1.3 | 302 | polyamines | CR/*P. putida* | Yes (22) | No | Yes (9) | dCACHE_1 | (1) |
| 3lib | Z3 | 28.0 | 21 | 2.2 | 280 | unknown | HK^b^*/Methanosarcina mazei* | Yes (20) | No | No | dCACHE_1 | (2) |
| 3li8 | Z2 | 27.2 | 21 | 2.3 | 280 | unknown | HK*/*[*M. mazei*](https://www.rcsb.org/pdb/search/smartSubquery.do?smartSearchSubtype=TreeEntityQuery&t=1&n=2209) | Yes (20) | No | No | dCACHE_1 | (2) |
| 4wy9 | Tlp1 | 23.6 | 16 | 4.0 | 298 | unknown | CR*/C. jejuni* | Yes (16) | Yes (2/-) | No | dCACHE_1 | (3) |
| 3lic | Z6 | 20.7 | 20 | 3.9 | 281 | unknown | HK*/*[*S. oneidensis*](https://www.rcsb.org/pdb/search/smartSubquery.do?smartSearchSubtype=TreeEntityQuery&t=1&n=70863) | Yes (7) | No | No | dCACHE_1 | (2) |
| 5ave | Mlp37 | 19.5 | 14 | 3.1 | 251 | L-Ser, taurine | CR/*V. cholerae* | No | No | No | dCACHE_1 | (4) |
| 5ltx | PctA | 19.3 | 18 | 2.7 | 252 | amino acids | CR/*P. aeruginosa* | No | No | No | dCACHE_1 | To be published |
| 5ere |  | 19.3 | 12 | 2.8 | 239^d^ | cytosine^e^ | Novel receptor*/*  [*D.*](https://www.rcsb.org/pdb/search/smartSubquery.do?smartSearchSubtype=TreeEntityQuery&t=1&n=45663) *retbaense* | No | No | No | Peripla_BP (CL0144) | To be published |
| 3lif | Z16 | 18.4 | 12 | 3.0 | 258 | unknown | HK/*R. palustris* | No | No | No | dCACHE1 | (2) |
| 5ltv | PctC | 18.3 | 22 | 2.8 | 255 | amino acids/GABA | CR/*P. aeruginosa* | No | No | No | dCACHE_1 | To be published |
| 5lt9 | PctB | 18.1 | 18 | 3.4 | 251 | amino acids | CR/*P. aeruginosa* | No | No | No | dCACHE_1 | To be published |
| 3lid | Z8 | 17.4 | 7 | 4.2 | 293 | unknown | HK/*V. parahaemolyticus* | No | Yes (-/3) | No | dCACHE_1 | (2) |
| 4xmq | Tlp3/  Ccml | 17.0 | 14 | 3.5 | 255 | amino acids, succinate, malate, fumarate, purine, thiamine | CR/*C. jejuni* | No | Yes (-/3) | No | dCACHE_1 | (5) |
| 2zbb | smDctB | 15.4 | 9 | 3.3 | 280 | dicarboxylic acids | HK/[[*S.*](https://www.rcsb.org/pdb/search/smartSubquery.do?smartSearchSubtype=TreeEntityQuery&t=1&n=266834) *meliloti*](https://www.rcsb.org/pdb/search/smartSubquery.do?smartSearchSubtype=TreeEntityQuery&t=1&n=266834) | No | Yes (2/3) | No | none | To be published |
| 3t4k | AHK4 | 15.3 | 11 | 3.5 | 281 | cytokinins | HK/*A.* *thaliana* | Yes (7) | No | No | CACHE-like  (CL0165) | (6) |
| 3by9 | vcDctB | 15.3 | 12 | 3.7 | 269 | dicarboxylic acids | HK/*V. cholerae* | No | Yes (2/3) | No | dCACHE_1 | (7) |

^a^ size between tm regions as determined by DAS (8), ^b^ HK: histidine kinase, CR: chemoreceptor, ^c^ size in amino acids is shown in brackets, ^d^ part of cytosolic receptor, LBD size determined by structural alignment, ^e^ cytosine present in the binding pocket in the membrane distal module.

**References**

1. Gavira JA, Ortega A, Martín-Mora D, Conejero-Muriel MT, Corral-Lugo A, Morel B, Matilla MA, Krell T. 2018. J Mol Biol 430:1950-1963.

2. Zhang Z, Hendrickson WA. 2010. J Mol Biol 400:335-53.

3. Machuca MA, Liu YC, Beckham SA, Gunzburg MJ, Roujeinikova A. 2016. J Struct Biol doi:10.1016/j.jsb.2016.02.019.

4. Nishiyama S, Takahashi Y, Yamamoto K, Suzuki D, Itoh Y, Sumita K, Uchida Y, Homma M, Imada K, Kawagishi I. 2016. Sci Rep 6:20866.

5. Liu YC, Machuca MA, Beckham SA, Gunzburg MJ, Roujeinikova A. 2015. Acta Crystallogr D Biol Crystallogr 71:2127-36.

6. Hothorn M, Dabi T, Chory J. 2011. Nat Chem Biol 7:766-8.

7. Cheung J, Hendrickson WA. 2008. J Biol Chem 283:30256-65.
