# Supplementary material for: High-Affinity Chemotaxis to Histamine Mediated by the TlpQ Chemoreceptor of the Human Pathogen Pseudomonas aeruginosa
Source: mBio. 2018 Nov 13;9(6):e01894-18. doi: 10.1128/mBio.01894-18 (PMC6234866; doi:10.1128/mBio.01894-18)
Supplement: TABLE S2 [file mbo006184178st2.docx]

**Table S2.**

| Plasmid | Primer Name | Sequence (5’ to 3’)^a^ | Endonuclease |
| --- | --- | --- | --- |
| pK18*mobsacB-pctA* | NM-PctA-Up-F | AT**GAATTC**AACAGCCAGAGCAGGAAAG | EcoRI |
|  | NM-PctA-Up-R | CAT**ACTAGT**CATGTTGTATTGCGTCCAGGAG | SpeI |
|  | NM-PctA-Down-F | CAT**ACTAGT**CAGCTTCAAGATCTGAGACCG | SpeI |
|  | NM-PctA-Down R | ACAT**GTCGAC**TGACCGGAGAGAAACTGAG | SalI |
| pK18*mobsacB*-*tlpQ* | NM-TlpQ-Up-F | GGT**CTCGAG**TATCCTTGACTTCGGCATCAG | XhoI |
|  | NM-TlpQ-Up-R | GAC **GAATTC** CCATTCTTGACGACGGTC | EcoRI |
|  | NM-TlpQ-Down-F | GAA**GAATTC**GGCCTGATGGAACAGTTCAG | EcoRI |
|  | NM-TlpQ-Down-R | ATG**GGATCC**GTGTGCAGTGGATGGGTCTG | BamHI |
| pK18*mobsacB-pctABC,orf1* | NM-PctABC-Orf1-Up-F | CTTTA**CTGCAG**AGACCCCTGCTATGCGTCC | PstI |
|  | NM-PctABC-Orf1-Up-R | AGAAC**GGATCC**CCTGGCAAAAGCCTCAAGAG | BamHI |
|  | NM-PctABC-Orf1-Down-F | GAACA**GGATCC**CAAGCTCAACGGAGGAAG | BamHI |
|  | NM-PctABC-Orf1-Down-R | GAAGT**GAATTC**CGAGGATACGCTCAGGAAGAAG | EcoRI |
| pK18*mobsacB-orf1-pctC* | NM-Orf1-PctC Up-F | CGAT**GAATTC**GGTACGTTGG | EcoRI |
|  | NM-Orf1-PctC-Up-R | AA**CTCGAG**TCCAATTCGACGGTGAAGAG | XhoI |
|  | NM-Orf1-PctC-Down-F | AA**CTCGAG**TTGCGACATCCATTCATCCTC | XhoI |
|  | NM-Orf1-PctC-Down-R | AA**GGATCC**GGCAAGTCCGGAAGCTTTAG | BamHI |
| pPctC | Comp-PctC-F | AA**GAATTC**GTGGCTCTGATTGCCGTATTG | EcoRI |
|  | Comp-PctC-R | AA**GAGCTC**TGTCCAATTCGACGGTGAAGA | SacI |
| pET28-b-TlpQ | TlpQ-LBD-F | CTGGTCGGCCTTTCGGTCTAC**CATATG** | NdeI |
|  | TlpQ-LBD-R | TGGGTCGAACT**GGATCC**CGGCCT | BamHI |

^a^Sequences recognized by restriction enzymes are bolded.
