# Supplementary material for: High-Affinity Chemotaxis to Histamine Mediated by the TlpQ Chemoreceptor of the Human Pathogen Pseudomonas aeruginosa
Source: mBio. 2018 Nov 13;9(6):e01894-18. doi: 10.1128/mBio.01894-18 (PMC6234866; doi:10.1128/mBio.01894-18)
Supplement: TABLE S3 [file mbo006184178st3.docx]

**Table S3.**

| Protein | TlpQ-LBD |
| --- | --- |
| Ligand | histamine |
| **PDB identifier** | 6fu4 |
| **Data collection** |  |
| Beam line | ID23-2 (ESRF) |
| Space Group | P 64 |
| Cell dimensions a, b, c (Å) | 81.62, 103.98, 147.45 |
| ASU | 4 |
| Resolution (Å) | 84.98 - 2.45 (2.54 - 2.45) |
| R*_merge_* (%) | 6.2 (36.9) |
| I/σ_I_ | 12.5 (2.9) |
| Completeness (%) | 98.12 (98.06) |
| Unique reflections | 46027 (4544) |
| Multiplicity | 3.9 (4.0) |
| CC(1/2) | 99.8 (82.6) |
| **Refinement** |  |
| Resolution (Å) | 84.98 - 2.45 |
| R*_work_*/R*_free_* (%) | 19.3/23.7 |
| No. atoms | 10086 |
| Protein | 9711 |
| Ligands | 70 |
| Water | 305 |
| B-factor (Å^2^) | 52.22 |
| R.m.s deviations |  |
| Bond lengths (Å) | 0.005 |
| Bond angles (^0^) | 1.08 |
| Ramachandran (%) |  |
| Favored | 97.86 |
| Outliers | 0.00 |
